# Dietary inflammatory index and metabolic syndrome in US children and adolescents: evidence from NHANES 2001–2018

**DOI:** 10.1186/s12986-022-00673-5

**Published:** 2022-06-13

**Authors:** Guhua Jia, Chieh-Chen Wu, Chun-Hsien Su

**Affiliations:** 1 Sports Teaching Department, Shanghai University of Medicine and Health Sciences, Shanghai, China; 2 Department of Exercise and Health Promotion, College of Kinesiology and Health, Chinese Culture University, Taipei, Taiwan; 3 Department of Exercise and Health Promotion, Graduate Institute of Sport Coaching Science, College of Kinesiology and Health, Chinese Culture University, No. 55, Hwa-Kang Road, Yang-Ming-Shan, Taipei City, 11114 Taiwan; 4 Department of Healthcare Information and Management, School of Health Technology, Ming Chuan University, Taipei, Taiwan

**Keywords:** Dietary inflammatory index (DII), Metabolic syndrome; children, Adolescent; exercise, National Health and Nutrition Examination Survey (NHANES)

## Abstract

**Background:**

An increasing number of children and adolescents are affected by metabolic syndrome (MetS). Dietary inflammatory index (DII) was associated with MetS in adult population. This study aimed to determine the associations between DII scores, MetS, and MetS components among children and adolescents.

**Methods:**

Data of children and adolescents in the National Health and Nutrition Examination Survey (NHANES) database 2001–2008 were obtained. DII was calculated for each participant based on the 24-h dietary recall interview. Univariate and multivariate logistic regression were conducted to determine the associations between DII, the other study variables and abnormal MetS components.

**Results:**

A total of 5,656 US children and adolescents (mean age = 15.49) in the 2001–2018 NHANES database were included. After adjusting for all confounders in the multivariate analysis, the top DII quartile was significantly and independently associated with increased odds of high blood pressure (BP) (aOR = 2.27, 95% CI: 1.02–5.07) as compared with the lowest DII quartile. DII in quartile 2, 3 or 4 were not significantly associated with increased odds of MetS, high waist circumference (WC), low high density lipoprotein-cholesterol (HDL-c), triglyceride (TG) or fasting plasma glucose (FPG) as compared with the lowest quartile. In stratified analysis by recommended physical activity level for children and adolescents, no significant association was observed between higher DII and MetS.

**Conclusions:**

Among US children and adolescents, high DII is associated with prevalent high BP but not MetS. The finding may contribute to future policymaking in promoting children’s health.

**Supplementary Information:**

The online version contains supplementary material available at 10.1186/s12986-022-00673-5.

## Background

Metabolic syndrome (MetS) is defined by a constellation of physiological, clinical, biochemical and metabolic factors, characterized by central obesity, hypertension, dyslipidemia, insulin resistance, and glucose intolerance [1]. As a result, MetS is strongly associated with an increased incidence of cardiovascular disease, type 2 diabetes and even with all-cause mortality [1]. It is postulated that these risks increasingly begin in childhood and likely lead to chronic diseases in adulthood [2, 3]. Currently, a progressively higher number of children and adolescents of developed and developing countries are affected by MetS, accompanied by increasing obesity and sedentary habits [4, 5]. Therefore, early identification of children at risk of developing MetS and appropriate intervention are paramount in order to minimize the future disease burden.

Diet is one of the central modulators of subclinical inflammation, which can be evaluated in humans through levels of markers including tumor necrosis factor-alpha (TNFα), high sensitivity C-reactive protein (hsCRP), or cell adhesion molecules [6]. The Western dietary pattern is associated with high hsCRP [7]. In contrast, the Mediterranean diet reduces inflammation and possibly protects adults and obese children against MetS [8–10].

Unveiling the link between dietary inflammatory potential and cardiometabolic risks may further benefit the control and prevention of cardiometabolic diseases. The DII score, developed in 2009 and updated in 2014, intends to evaluate the inflammatory potential of individual’s diet based on the balance of pro-and anti-inflammatory properties of its components, including macronutrients, vitamins, minerals, flavonoids, and specific food ingredients [11, 12]. DII scores are calculated and standardized to the global reference derived from eleven population around the world, allowing their use across distinct ethnic populations and patterns of diet [11, 12].

As inflammation may represent a triggering factor in the origin of MetS, [13, 14] there were emerging interests on directly linking DII with MetS among various population, both Eastern and Western [15–18]. However, most of the previous studies assessed the associations merely among the adult population, and evidence on the link between dietary inflammatory potential and MetS and its components among children and adolescents is scarce. Considering the health impact of MetS in pediatric population, accordingly, this study aimed to determine the associations between DII scores and MetS in children and adolescents using a large cohort of the national representative database, with the hypothesis that increased DII is associated with prevalent MetS and its components among this specific population.

## Methods

### Data source

This study used data from the National Health and Nutrition Examination Survey (NHANES) database, which was collected by the Centers for Disease Control and Prevention (CDC), National Center for Health Statistics (NCHS) in the USA. The survey is designed to evaluate the health and nutritional status of adults and children in the USA. It uses a complex, multistage design to collect and analyze data representative of the national, non-institutionalized population. A household interview and extensive examination in a mobile examination center (MEC), such as a physical examination, specialized measurements, and laboratory tests, were performed for participants in the NHANES. Such data is reliable and can be equated to a population-level assessment [19]. The NHANES was reviewed and approved through the NCHS Research Ethics Review Board, and each participant provided informed consent (https://www.cdc.gov/nchs/nhanes/irba98.htm). Additionally, all NHANES data released by the NCHS is de-identified, and remained anonymous during data analysis.

### Study population

The present study extracted data from nine study cycles in the NHANES database: 2001–2002, 2003–2004, 2005–2006, 2007–2008, 2009–2010, 2011–2012, 2013–2014, 2015–2016, 2017–2018). Children and adolescents aged 12–19 years who had complete information on the components of MetS and 24-h dietary recall data for computing DII were eligible to be included.

## Study variables

### Assessment of MetS and its components

We used a previously validated criteria developed by Jolliffe et al. for defining the adolescent (12 to 19 years old) MetS in the present analysis [20]. This criteria developed age- and sex-specific cut-points for WC, BP, HDL-c, TG and FPG level based on data from the Third National Health and Nutrition Examination Survey (NHANES III, 1988 to 1994) and the 1999–2000 and 2001–2002 NHANES surveys. In particular, age- and gender-specific growth curves were developed with the Lambda Mu Sigma method. At first, each MetS component growth curve was linked to the respective Adult Treatment Panel III (ATP) and International Diabetes Federation (IDF) criteria cut-points. Then, the adolescent cut-points were linked to adults' by defining the percentile corresponding to the adult cut-point and regressing it backward into adolescence [20]. Details of the cut-points for each MetS criteria could were documented in Additional file 1: Table S1. In the present analysis, we followed the ATP criteria to define MetS as fulfilling three of the five criteria.

### Assessment of DII

In calculating each individual’s DII, information of dietary intake for the nutrients and food items were derived from the NHANES 24-h recall data. Compared to the global reference database, a Z-score for each participant's food parameters was calculated by subtracting the global mean with the reported amount and dividing this value by the standard deviation. They were then converted to a proportion to minimize the effects of outliers. The standardized proportion was centered by doubling and subtracting one and then multiplied by the inflammatory effect score of each food parameter and summed to obtain an overall DII score for every participant in the study [11]. Higher DII scores represents a more proinflammatory diet, namely, a greater proinflammatory potential of the diet. In the present study, DII were categorized into quartiles, in which the highest quartile (Q4) represents the greatest proinflammatory potential. The information of vitamin D intake was not consistently reported by the NHANES across the study cycles and was omitted from the present analyses, in which 26 food parameters were included totally.

### Covariates

Demographic data, including age, gender, race, family income to poverty ratio, and education level, were obtained through in-person interviews by trained interviewers using the Family and Sample Person Demographics questionnaires and the Computer-Assisted Personal Interviewing (CAPI) system (Confirmit Corp. New York, USA). Collected data were weighted following the NHANES protocol.

Smoke exposure status was classified into three groups: not exposed, second-hand smoke exposure, and active smoker, identified by individual’s response to the questionnaires related to smoking combined with their serum nicotine level in the biochemical profile of the NHANES. Not exposed: no self-report smoking within past 30 days and serum cotinine < 0.05 μg/L; second-hand smoke exposure: no self-report smoking within past 30 days and with a serum cotinine level at 0.05–15 μg/L; active smoker: self-report smoking within past 30 days or having a serum cotinine level at ≥ 15 μg/L.

In estimating physical activity, the product of weekly time spent in each activity reported by the participant multiplied by the metabolic equivalent of task (MET) value for that activity were summed, yielding a MET-h index. One MET represents the energy expenditure of 1 kcal/kg body weight per hour. The current physical activity guideline recommends an average daily engagement in 60 min of moderate-to-vigorous physical activity (MVPA) for children and adolescents. Accordingly, we have categorized participants’ physical activity level into ideal (at or above recommendation) and non-ideal (below recommendation) [21].

Sedentary time was assessed by the NHANES through the individual’s daily hours of TV, video, or computer use according to the in-person interview data and was divided into three categories: < 3, 3–6, and ≥ 6 h.

### Statistical analysis

The NHANES uses a complex survey design to assure national representation, wherein sampling weights (WTMEC2YR), pseudo-stratum (SDMVSTRA), and pseudo-cluster (SDMVPSU) provided by the NHANES were applied in all analyses as guided by NCHS. [23] Continuous variables are displayed as mean and standard error (SE), and categorical variables are presented as unweighted counts (weighted %). Linear regression analysis tested differences in continuous variables, and categorical variables were assessed using a Wald chi-square test. Univariate and multivariate logistic regression models were performed to present the associations between the study variables, MetS, and its principal components. Data were shown as adjusted odds ratio (aOR) and 95% confidence interval (CI). Variables that showed significant values in the univariate analysis were adjusted in multivariate analyses. All statistical assessments were two-sided and evaluated with a significance of 0.05. Statistical analyses were performed using SAS statistical software (version 9.4, SAS Inc., Cary, NC, USA).

## Results

**Fig. 1:**
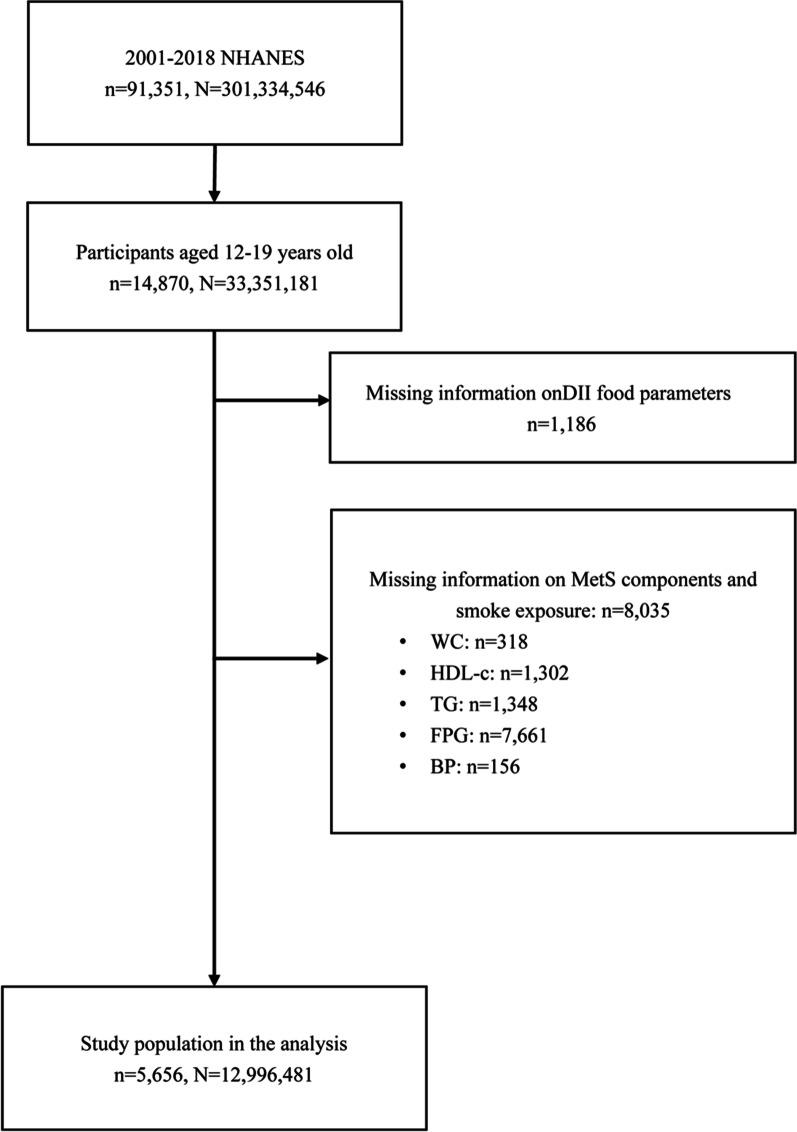
Flowchart of study selection

Figure 1 summarizes the process of cohort selection. After excluding the participants of missing information on the parameters for calculating DII or the components in defining MetS, a total of 5656 children and adolescent participants aged 12 to 19 years old in the NHANES during 2001 and 2018 were included as the primary cohort for further analyses. This final analytic sample was equivalent to a population-based sample size of 12,996,481 subjects in the US, using discharge weights provided by NHANES (Fig. 1**).**

Characteristics of the study participants are shown in Table 1, grouped into DII quartiles. The mean (SE) age of the study population was 15.49 (0.05). There were 2,937 (51.77%) males and 2,712 (48.23%) females. The mean (SE) DII score was -1.46 (0.02) in the overall study population. The ranges of DII scores in quartiles were < −1.95, −1.95 to −1.31, −1.31 to −0.79, and ≥ −0.79 from quartile 1 (Q1) to quartile 4 (Q4), respectively. The frequencies of prevalent high WC and low HDL-c differed significantly between DII quartiles. Children in the DII Q3 group had the highest proportion of high WC (29.96%, *p* < 0.001) compared with the other DII groups. Children in the DII Q4 group had the highest proportion of low HDL-C (30.04%, *p* = 0.004) compared with the other DII groups. Age, sex, BMI, race, family income level, energy intake, smoke exposure, and physical activity level showed a significant difference between DII quartiles (*p* = 0.004 for family income level and *p* < 0.001 for the others). In specific, as compared with DII Q1 to Q3, children in the DII Q4 group were younger (mean: 15.24 years old), of greater BMI (24.23 kg/m2), with lower energy intake (1384.64 kcal) and lower physical activity (2981.17 MET-min/week). DII Q4 group also had greater proportions of female (28.86%), non-Hispanic Black (28.88%), children from low-income family (26.35%), with secondhand smoke exposure, and active smoker (31.03% and 27.56%). (Table 1).

**Table 1:** Characteristics of study population

	Overall n = 5,656	DII Q1 (< −1.95) n = 1414	DII Q2 (−1.95 ~ −1.31) n = 1414	DII Q3 (−1.31 ~ −0.79) n = 1414	DII Q4 (> = −0.79) n = 1414	*p* value
DII score	−1.46 (0.02)	−2.63 (0.02)	−1.61 (0.01)	−1.05 (0.00)	−0.51 (0.01)	** < 0.001**^**a**^
MetS	394 (6.83%)	82 (23.39%)	94 (25.40%)	102 (22.48%)	116 (28.73%)	0.301^b^
**MetS components**						
High WC	1330 (22.87%)	259 (19.65%)	304 (23.69%)	386 (29.96%)	381 (26.70%)	** < 0.001 **^**b**^
Low HDL-c	1308 (24.57%)	265 (21.63%)	314 (24.15%)	325 (24.17%)	404 (30.04%)	**0.004 **^**b**^
High TG	565 (10.61%)	151 (26.24%)	144 (25.70%)	134 (22.77%)	136 (25.30%)	0.814^b^
High FPG	943 (17.86%)	233 (25.80%)	241 (26.58%)	236 (22.26%)	233 (25.35%)	0.440^b^
High BP (mmHg)	331 (4.95%)	71 (22.08%)	95 (28.87%)	94 (27.87%)	71 (21.18%)	0.427^b^
**Demography**						
Age (years)	15.49 (0.05)	15.73 (0.09)	15.63 (0.09)	15.35 (0.08)	15.24 (0.09)	** < 0.001**^a^
Sex						** < 0.001 **^**b**^
Male	2937 (51.77%)	913 (32.59%)	753 (25.27%)	681 (22.20%)	590 (19.94%)	
Female	2712 (48.23%)	501 (17.71%)	659 (25.75%)	732 (27.69%)	820 (28.86%)	
BMI (kg/m^2^)	23.78 (0.11)	23.28 (0.19)	23.73 (0.19)	23.92 (0.20)	24.23 (0.18)	** < 0.001**^a^
Race						** < 0.001 **^**b**^
Non-Hispanic White	1599 (58.97%)	407 (25.18%)	394 (25.44%)	398 (24.91%)	400 (24.47%)	
Non-Hispanic Black	1648 (14.07%)	328 (20.12%)	399 (24.91%)	443 (26.09%)	478 (28.88%)	
Mexican American	1517 (13.33%)	418 (28.88%)	415 (27.77%)	351 (23.42%)	333 (19.93%)	
Other	885 (13.64%)	261 (28.47%)	204 (24.15%)	221 (24.70%)	199 (22.68%)	
Family income level						**0.004 **^**b**^
Not poor	3595 (77.62%)	913 (25.37%)	924 (26.54%)	888 (24.50%)	870 (23.60%)	
Poor	1649 (22.38%)	392 (26.16%)	386 (21.66%)	429 (25.83%)	442 (26.35%)	
**Lifestyle**						
Energy intake (kcal)	2178.13 (19.72)	3162.66 (46.45)	2246.48 (26.47)	1875.18 (18.46)	1384.64 (19.82)	** < 0.001 **^**a**^
Smoke exposure						** < 0.001**^**b**^
Not exposed	2577 (48.57%)	719 (28.56%)	699 (27.72%)	639 (25.41%)	520 (18.30%)	
Secondhand smoke exposed	2136 (33.85%)	441 (21.65%)	488 (23.16%)	548 (24.15%)	659 (31.03%)	
Active smoker	936 (17.58%)	254 (23.94%)	225 (23.86%)	226 (24.64%)	231 (27.56%)	
Sedentary time (hours)						0.071^b^
< 3	1708 (39.01%)	417 (25.18%)	397 (24.52%)	418 (23.80%)	476 (26.50%)	
3–6	2257 (47.88%)	549 (24.14%)	604 (27.12%)	578 (27.10%)	526 (21.64%)	
> 6	550 (13.11%)	140 (24.63%)	127 (21.13%)	136 (24.26%)	147 (29.98%)	
Physical activity (MET-min/week)	3263.04 (73.84)	3691.73 (118.65)	3283.25 (133.91)	3062.45 (124.70)	2981.17 (150.30)	** < 0.001 **^**a**^
Physical activity, categories^c^						** < 0.044 **^**b**^
Not ideal	3547 (62.71%)	823 (23.24%)	897 (25.96%)	900 (25.96%)	927 (24.84%)	
Ideal	2109 (37.29)	591 (27.61%)	517 (25.06%)	514 (23.67%)	487 (23.66%)	

Unweighted counts (weighted %) are presented for categorical variables and mean (SE) were presented for continuous data

Significant variables are shown in bold

DII, dietary inflammatory index; FPG, fasting plasma glucose; MetS, metabolic syndrome; WC, waist circumference; HDL-c, high-density lipoprotein cholesterol; TG, triglyceride; BP, blood pressure; MET, Metabolic Equivalent of Task; Q, quartile

^a^Using proc surveyreg procedure

^b^Wald chi-square test

^**c**^Ideal physical activity: ≥ 180 MET-min/day (i.e., 60 min/day moderate-to-vigorous physical activity); not ideal physical activity: < 180 MET-min/day

Table 2 shows the associations between the study variables and MetS, high WC, and low HDL-c. In the univariate analysis, it was found that DII Q3 and Q4 was significantly associated with a higher odds of high WC (OR = 1.77, 95% CI: 1.14–2.23; 1.57, 95% CI: 1.23, 2.00, respectively) as compared with the lowest quartile. DII Q4 was significantly associated with increased odds of low HDL-c (OR = 1.66; 95% CI: 1.26–2.17) as compared with the lowest quartile. However, after adjusting for all relevant confounders in the multivariate analysis, they became statistically insignificant. (Table 2).

**Table 2:** Associations between study variables, prevalent MetS, high WC and low HDL-c

	MetS		High WC		Low HDL-c	
	Univariate analysis OR (95% CI)	Multivariate analysis aOR (95% CI)	Univariate analysis OR (95% CI)	Multivariate analysis aOR (95% CI)	Univariate analysis OR (95% CI)	Multivariate analysis aOR (95% CI)
**DII score**						
DII Q1	Ref	Ref	Ref	Ref	Ref	Ref
DII Q2	1.09 (0.72, 1.64)	1.01 (0.62, 1.63)	1.26 (0.97, 1.63)	0.78 (0.49, 1.26)	1.15 (0.88, 1.49)	1.04 (0.77, 1.40)
DII Q3	0.98 (0.66, 1.46)	0.80 (0.51, 1.24)	**1.77 (1.41**, **2.23)**	1.41 (0.93, 2.13)	1.19 (0.92, 1.54)	1.02 (0.75, 1.40)
DII Q4	1.31 (0.93, 1.85)	1.09 (0.73, 1.62)	**1.57 (1.23**, **2.00)**	0.82 (0.47, 1.45)	**1.66 (1.26**, **2.17)**	1.39 (0.94, 2.06)
**Demography**						
BMI (kg/m^2^)	**1.23 (1.20**, **1.25)**	**1.23 (1.20**, **1.26)**	**1.77 (1.71**, **1.84)**	**1.86 (1.78**, **1.94)**	**1.13 (1.11**, **1.15)**	**1.14 (1.12**, **1.16)**
**Race**						
Non-Hispanic White	Ref	Ref	Ref	Ref	Ref	Ref
Non-Hispanic Black	0.84 (0.57, 1.24)	**0.36 (0.23**, **0.57)**	1.13 (0.93, 1.38)	**0.32 (0.23**, **0.46)**	**0.62 (0.49**, **0.79)**	**0.44 (0.34**, **0.56)**
Mexican American	**1.91 (1.38**, **2.65)**	**1.60 (1.11**, **2.32)**	**1.53 (1.25**, **1.87)**	0.86 (0.62, 1.20)	1.16 (0.92, 1.45)	0.99 (0.77, 1.27)
Other	0.88 (0.60, 1.29)	0.97 (0.63, 1.49)	1.01 (0.77, 1.31)	0.82 (0.54, 1.24)	0.91 (0.70, 1.19)	0.94 (0.71, 1.24)
**Family income level**						
Not poor	Ref	–	Ref	Ref	Ref	–
Poor	1.26 (0.93, 1.71)	–	**1.41 (1.17**, **1.69)**	**1.43 (1.01**, **2.03)**	1.13 (0.93, 1.37)	–
**Lifestyle**						
Energy intake (kcal)	1.00 (1.00, 1.00)	–	**1.00 (1.00**, **1.00)**	**1.00 (1.00**, **1.00)**	**1.00 (1.00**, **1.00)**	1.00 (1.00, 1.00)
Smoke exposure						
Not exposed	Ref	Ref	Ref	Ref	Ref	–
Secondhand smoke exposed	**1.66 (1.22**, **2.24)**	**1.49 (1.04**, **2.13)**	**1.31 (1.07**, **1.61)**	0.86 (0.60, 1.24)	1.02 (0.85, 1.23)	–
Active smoker	**1.71 (1.18**, **2.48)**	1.45 (0.75, 1.75)	1.03 (0.78, 1.36)	**0.27 (0.17**, **0.44)**	1.15 (0.88, 1.50)	–
**Sedentary time (hours)**						
< 3	Ref	Ref	Ref	Ref	Ref	–
3–6	**2.00 (1.41**, **2.84)**	1.42 (0.90, 2.23)	**1.49 (1.22**, **1.82)**	1.12 (0.81, 1.54)	1.01 (0.84, 1.21)	–
> 6	**2.72 (1.85**, **4.00)**	1.43 (0.91, 2.24)	**1.87 (1.48**, **2.38)**	0.87 (0.52, 1.44)	0.99 (0.72, 1.35)	–
Physical activity (MET-min/week)	1.00 (1.00, 1.00)	–	**1.00 (1.00**, **1.00)**	–	**1.00 (1.00**, **1.00)**	–
**Physical activity (MET-min/week)**, ** categories**^**a**^						
Not ideal	Ref	Ref	Ref		Ref	
Ideal	**0.73 (0.53**, **0.99)**	0.72 (0.47, 1.10)	0.82 (0.67, 1.00)		1.00 (0.84, 1.19)	

Significant values are shown in bold. Missing values in each variable were excluded from the analyses

*DII* dietary inflammatory index, *FPG* fasting plasma glucose, *MetS* metabolic syndrome, *WC* waist circumference, *HDL-c* high-density lipoprotein cholesterol, *TG* triglyceride, *BP* blood pressure, *MET* metabolic equivalent of task, *Q* quartile

^a^Ideal physical activity: ≥ 180 MET-min/day (i.e., 60 min/day moderate-to-vigorous physical activity); Not ideal physical activity: < 180 MET-min/day

Table 3 shows the associations between study variables and high TG, high FPG, and high BP. In the univariate analysis, DII in quartiles was not significantly associated with any of the metabolic components. However, after adjusting for relevant confounders in the multivariate analysis, DII Q4 was significantly and independently associated with increased odds of high BP (aOR = 2.27, 95% CI: 1.02–5.07) as compared with the lowest DII quartile (Table 3).

**Table 3:** Associations between study variables, high TG, high FPG and high BP

	High TG		High FPG		High BP	
	Univariate analysis OR (95% CI)	Multivariate analysis aOR (95% CI)	Univariate analysis OR (95% CI)	Multivariate analysis aOR (95% CI)	Univariate analysis OR (95% CI)	Multivariate analysis aOR (95% CI)
**DII score**						
DII Q1	Ref	Ref	Ref	Ref	Ref	Ref
DII Q2	0.87 (0.56, 1.35)	0.95 (0.69, 1.31)	1.03 (0.80, 1.33)	1.04 (0.81, 1.35)	1.32 (0.87, 2.01)	2.09 (0.88, 4.95)
DII Q3	0.83 (0.53, 1.30)	0.81 (0.56, 1.17)	0.86 (0.67, 1.10)	0.87 (0.68, 1.12)	1.31 (0.84, 2.05)	2.17 (0.89, 5.28)
DII Q4	0.92 (0.61, 1.41)	0.92 (0.67, 1.26)	1.04 (0.82, 1.31)	1.03 (0.81, 1.31)	1.01 (0.65, 1.56)	**2.27 (1.02**, **5.07)**
**Demography**						
BMI	**1.10 (1.09**, **1.12)**	**1.11 (1.09**, **1.13)**	**1.03 (1.01**, **1.05)**	**1.03 (1.01**, **1.05)**	**1.10 (1.08**, **1.12)**	**1.12 (1.09**, **1.15)**
**Race**						
Non-Hispanic White	Ref	Ref	Ref	Ref	Ref	Ref
Non-Hispanic Black	**0.35 (0.25**, **0.50)**	**0.23 (0.16,0.34)**	0.78 (0.58, 1.06)	**0.73 (0.54**, **0.98)**	**2.44 (1.75**, **3.42)**	**1.13 (0.71**, **1.81)**
Mexican American	1.16 (0.90, 1.49)	**0.95(0.73**, **1.24)**	**1.61 (1.26**, **2.07)**	**1.56 (1.22**, **1.99)**	0.92 (0.59, 1.44)	0.58 (0.31, 1.09)
Other	0.95 (0.69, 1.30)		1.17 (0.88, 1.57)	1.16 (0.87, 1.55)	1.00 (0.63, 1.58)	0.46 (0.16, 1.35)
**Family income level**						
Not poor	Ref	Ref	Ref	–	Ref	–
Poor	**1.30 (1.02**, **1.66)**	1.30 (0.99, 1.69)	1.13 (0.89, 1.45)	–	1.10 (0.83, 1.46)	–
**Lifestyle**						
Energy intake	1.00 (1.00, 1.00)	–	1.00 (1.00, 1.00)	–	**1.00 (1.00**, **1.00)**	**1.00 (1.00**, **1.00)**
**Smoke exposure**						
Not exposed	Ref	Ref	Ref	–	Ref	Ref
Secondhand smoke exposed	1.20 (0.93, 1.54)	1.18 (0.90, 1.54)	1.09 (0.88, 1.35)	–	**1.66 (1.14**, **2.43)**	0.99 (0.56, 1.73)
Active smoker	**1.66 (1.23**, **2.23)**	1.35 (0.98, 1.87)	1.21 (0.91, 1.60)	–	1.23 (0.77, 1.95)	0.69 (0.34, 1.44)
**Sedentary time**						
< 3	Ref	Ref	Ref	Ref	Ref	–
3–6	**1.37 (1.04**, **1.81)**	1.20 (0.89, 1.63)	**1.32 (1.04**, **1.69)**	1.26 (0.98, 1.62)	1.17 (0.81, 1.68)	–
> 6	**1.58 (1.08**, **2.31)**	1.27 (0.82, 1.96)	**2.10 (1.47**, **2.99)**	**2.01 (1.38**, **2.92)**	1.21 (0.68, 2.16)	–
Physical activity (MET-min/week)	1.00 (1.00, 1.00)	–	1.00 (1.00, 1.00)	–	**1.00 (1.00**, **1.00)**	**1.00 (1.00**, **1.00)**
**Physical activity (MET-min/week), categories**^**a**^						
Not ideal	Ref		Ref		Ref	
Ideal	0.88 (0.70, 1.11)		1.11 (0.90, 1.38)		**0.67 (0.46**, **0.98)**	

Significant values are shown in bold. Missing values in each variable were excluded from the analyses

*DII* dietary inflammatory index, *FPG* fasting plasma glucose, *MetS* metabolic syndrome, *WC* waist circumference, *HDL-c* high-density lipoprotein cholesterol, *TG* triglyceride, *BP* blood pressure, *MET* metabolic equivalent of task, *Q* quartile

^a^Ideal physical activity: ≥ 180 MET-min/day (i.e., 60 min/day moderate-to-vigorous physical activity); Not ideal physical activity: < 180 MET-min/day

Table 4 shows the associations between DII in quartiles and MetS stratified by the children’s physical activity level. After adjustments, no significant association between higher DII and MetS among physical activity subgroups was observed (Table 4).

**Table 4:** Associations between DII and MetS, stratified by physical activity level

	DII Q2	DII Q3	DII Q4
	aOR (95% CI)	aOR (95% CI)	aOR (95% CI)
**Physical activity**^**a**^			
Not ideal (n = 3541)	0.68 (0.38,1.20)	0.73 (0.40,1.31)	0.89 (0.55,1.46)
Ideal (n = 2106)	1.65 (0.79,3.43)	0.92 (0.45,1.88)	1.38 (0.74,2.59)

Adjusted for BMI, race, smoke exposure and sedentary time

*DII* dietary inflammatory index, *MetS* metabolic syndrome, *MET* metabolic equivalent of task, *Q* quartile

^a^Ideal physical activity: ≥ 180 MET-min/day (i.e., 60 min/day moderate-to-vigorous physical activity); not ideal physical activity: < 180 MET-min/day

## Discussion

The present study aimed to determine the associations between DII and MetS among children and adolescents. After adjusting for relevant confounders, US children and adolescents aged 12–19 years old who had highest DII were more likely to have high BP than those with low DII. However, higher DII seemed not significantly associated with prevalent MetS.

For adult population, more and more shreds of evidence displayed the associations between DII and MetS. For example, a previous study suggested the association between DII scores and MetS among 17,689 adults in the US and reinforced the view that diet plays a vital role in the occurrence of cardiovascular diseases [22]. Another study with 9,291 Korean adults showed that the top DII quartile (Q4) was positively associated with MetS prevalence in men and postmenopausal women [15]. In that study, the top DII quartile was also positively associated with prevalent hyperglycemia in men and central obesity in postmenopausal women. Further, the results of a systematic review by Yi et al. revealed significant positive associations of higher DII with MetS, abdominal obesity, high blood pressure, hyperglycemia, and hypertriglyceridemia [18].

Dietary pattern analysis, rather than a single nutrient or food intake, can be used to investigate associations between food intake and diseases in specific populations [9]. Some previous studies analyzing the dietary patterns of children and adolescents have been conducted in Europe and Australia [10, 23, 24], in which a relationship between the Westernized dietary pattern and increased risk of MetS was observed. A study in Korea reported white rice and kimchi consuming were associated with lower weight and higher HDL cholesterol, and oil and seasoned vegetable consuming were significantly associated with a lower weight, WC, and serum insulin level, whereas fast-food and soda consuming correlated with higher WC, serum insulin and BMI [25].

Despite the studies focusing on the DII and MetS in adults, and the dietary patterns in children cited above, evidence about the potential relationship between DII and MetS components among children is not sufficient. A previous cross-sectional study with 532 European adolescents indicated higher DII were associated with increased levels of various inflammatory markers such as TNF-α, IL-1, 2, IFN-γ, and vascular cell adhesion, providing a validation on the use of DII in adolescents [12]. A recent study by Kurklu et al. may be the only one in the medical literature assessing the role of DII on pediatric MetS, analyzing data of 343 adolescents and suggesting a significant association between higher DII scores and an increased risk of MetS along with several MetS components [26]. However, the single-center design and the small sample size in that study limit its results for further interpretation. To the best of our knowledge, the present analysis was the first to assess the potential associations between DII, MetS and its components in children and adolescents with a large, nationally representative data.

Benefits of physical exercise on metabolic health were reported by a long list of studies in the literature. One study using data during the NHANES 2007–2010 showed that leisure-time physical activity is associated with increases in longevity [21]. Another previous study highlighted the health benefits of physical activity against MetS in pediatric populations [27]. A previous meta-analysis indicated that physical exercise could improve the inflammatory state in obese children [28]. Other studies also suggested that physical activity might play a role in decreasing insulin resistance and MetS in children and adolescents [29, 30]. In the present study, stratified analysis was done by whether the physical activity level was ideal or not, but there seemed no significant association between DII and MetS among different physical activity level categories.

A previous clinical study found that the nutrition education was effective to improve the knowledge, dietary habits, and physical activity of the participants. Furthermore, the modification of the diet, i.e., higher intake of polyphenols (flavonoids and anthocyanins), fiber, polyunsaturated fatty acids (PUFA), PUFA n-3, and lower intake of saturated fatty acids (SFA) had a significant impact on the improvement of some MetS components before pharmacologic intervention [31]. Although no direct causal inference could be made based on the cross-sectional nature of the present analysis, our findings did highlight the relevance of inflammatory dietary pattern with abnormal BP among children and adolescents. Future longitudinal studies are needed to investigate whether a reduction in dietary components that have been linked to inflammation decreases MetS risk among this specific population.

### Strengths and limitation

The present study had several strengths. Firstly, data from NHANES were drawing from a large and diverse sample of non-institutionalized subjects, where the findings are likely generalizable to the overall U.S. population. Secondly, the MetS components were objectively measured and documented. Thirdly, various demographic and lifestyle factors were carefully adjusted. Fourthly, stratified analysis was performed to assess the potential modifying effect of physical activity in association between DII and MetS. There are also several limitations. It is a cross-sectional study, which causal inferences cannot be made. Dietary recall information and part of the covariates included are based on interviews and questionnaires; thus, inaccurate reporting or recall bias may have occurred. Physical activity levels and sedentary behavior were assessed through questionnaires but not objective measures such as accelerometer. Lastly, there may also be unknown confounders not included in the NHANES data.

## Conclusions

This study found associations between DII assessed pro-inflammatory diet and high BP in children and adolescent. The findings emphasize the relevance of healthy diet and components of MetS, and contribute to future policymaking and strategy optimizing in promoting children’ health.

## Supplementary Information

**Additional file 1**.** Supplementary Table 1**. Age specific cut-off points for MetS components of children and adolescents 12-19 years.

## Data Availability

The datasets analysed during the current study are available in this article.

## Abbreviations

MetS: Metabolic syndrome
TNFα: Tumor necrosis factor-alpha
hsCRP: High sensitivity C-reactive protein
NHANES: Nutrition Examination Survey
CDC: Centers for Disease Control and Prevention
NCHS: National Center for Health Statistics
MEC: Mobile examination center
