# Supplementary material for: Dietary inflammatory index and metabolic syndrome in US children and adolescents: evidence from NHANES 2001–2018
Source: Nutr Metab (Lond). 2022 Jun 13;19:39. doi: 10.1186/s12986-022-00673-5 (PMC9195322; doi:10.1186/s12986-022-00673-5)
Supplement: Supplementary file 1 — Additional file 1. Supplementary Table 1. Age specific cut-off points for MetS components of children and adolescents 12-19 years. [file 12986_2022_673_MOESM1_ESM.docx]

**Supplementary Table 1. Age specific cut-off points for MetS components of children and adolescents 12-19 years.**

|  | WC (cm) | BP (mm Hg) | | HDL-c (mmol/l) | TG (mmol/l) | FPG (mmol/l) |
| --- | --- | --- | --- | --- | --- | --- |
|  |  | SBP | DBP |  |  |  |
| **Male** |  |  | |  |  |  |
| Age (yrs) |  |  |  |  |  |  |
| 12 | 94.2 | 121 | 76 | 1.13 | 1.44 | 5.6 |
| 13 | 96.2 | 123 | 78 | 1.10 | 1.48 | 5.6 |
| 14 | 98.0 | 125 | 79 | 1.07 | 1.52 | 5.6 |
| 15 | 99.5 | 126 | 81 | 1.04 | 1.56 | 5.6 |
| 16 | 100.6 | 128 | 82 | 1.03 | 1.59 | 5.6 |
| 17 | 101.4 | 128 | 83 | 1.03 | 1.62 | 5.6 |
| 18 | 101.8 | 129 | 84 | 1.03 | 1.65 | 5.6 |
| 19 | 102.0 | 130 | 85 | 1.03 | 1.68 | 5.6 |
| **Female** |  |  | |  |  |  |
| Age (yrs) |  |  | |  |  |  |
| 12 | 79.5 | 121 | 80 | 1.25 | 1.60 | 5.6 |
| 13 | 81.3 | 123 | 82 | 1.25 | 1.53 | 5.6 |
| 14 | 82.9 | 125 | 83 | 1.26 | 1.46 | 5.6 |
| 15 | 84.2 | 126 | 84 | 1.26 | 1.44 | 5.6 |
| 16 | 85.2 | 128 | 84 | 1.27 | 1.46 | 5.6 |
| 17 | 86.2 | 128 | 85 | 1.27 | 1.53 | 5.6 |
| 18 | 87.0 | 129 | 85 | 1.28 | 1.61 | 5.6 |
| 19 | 87.7 | 130 | 85 | 1.29 | 1.68 | 5.6 |

WC, waist circumference; BP, blood pressure; SBP, systolic blood pressure; DBP, diastolic blood pressure; HDL-c, high density lipoprotein-cholesterol; TG, triglyceride; FPG, fasting plasma glucose.
